# Exploring long-wave infrared transmitting materials with *A*_*x*_*B*_*y*_ form: First-principles gene-like studies

**DOI:** 10.1038/srep21912

**Published:** 2016-02-23

**Authors:** Jia-Ren Du, Nian-Ke Chen, Xian-Bin Li, Sheng-Yi Xie, Wei Quan Tian, Xian-Yin Wang, Hai-Ling Tu, Hong-Bo Sun

**Affiliations:** 1 State Key Laboratory on Integrated Optoelectronics, College of Electronic Science and Engineering, Jilin University, Changchun 130012, China; 2 College of Chemistry and Chemical Engineering, Huxi Campus, Chongqing University, Chongqing 401331, China; 3 General Research Institute for Nonferrous Metals, Beijing 100088, China

## Abstract

Long-wave infrared (8–12 μm) transmitting materials play critical roles in space science and electronic science. However, the paradox between their mechanical strength and infrared transmitting performance seriously prohibits their applications in harsh external environment. From the experimental view, searching a good window material compatible with both properties is a vast trail-and-error engineering project, which is not readily achieved efficiently. In this work, we propose a very simple and efficient method to explore potential infrared window materials with suitable mechanical property by first-principles gene-like searching. Two hundred and fifty-three potential materials are evaluated to find their bulk modulus (for mechanical performance) and phonon vibrational frequency (for optical performance). Seven new potential candidates are selected, namely TiSe, TiS, MgS, CdF_2_, HgF_2_, CdO, and SrO. Especially, the performances of TiS and CdF_2_ can be comparable to that of the most popular commercial ZnS at high temperature. Finally, we propose possible ranges of infrared transmission for halogen, chalcogen and nitrogen compounds respectively to guide further exploration. The present strategy to explore IR window materials can significantly speed up the new development progress. The same idea can be used for other material rapid searching towards special functions and applications.

Infrared radiation (IR) is with longer wavelengths than those of visible light, extending from the red edge of the visible spectrum at 700 nm to 1 mm. It has been broadly used in industrial, scientific, medical and military fields. Besides the extensively focused IR absorption material, IR transmitting material is another critical component for sensing and detecting system^1^,^2^,^3^. Generally, IR imaging takes place in the wavelength between 0.75–14 μm, which contains the “fingerprint region”^4^,^5^, where a large number of molecules undergo strong characteristic vibration transitions, leaving the distinctive spectral absorption fingerprints. The propagation of IR is limited by the absorption and the scatter in atmosphere. According to the transmission spectrum in atmosphere, two high transmission regions, known as the transmission windows, are the mid-wave infrared (MWIR) window (from 3 to 5 μm) and the long-wave infrared (LWIR) window (from 8 to 12 μm)^2^,^5^,^6^,^7^,^8^. Such windows are quite useful in many scientific and technical areas. For example, the transmitting materials have been applied in these window regions to detect the small trace of the environmental or toxic vapors with a parts-per-billion sensitivity^5^,^9^. Generally, the LWIR transmitting materials can play a critical role in infrared surveillance, searching, recognition, guidance, reconnaissance, navigation and imaging due to their broader sensitivity than that of the MWIR window materials. Also, the LWIR window material can also facilitate a safer inspection of the energized electrical equipment^10^.

An ideal IR-window material usually possesses a high (and wide) transmittance, and a good mechanical strength including high durability, hardness, chemical stability, wind/rain-erosion resistance and thermal shock resistance^2^,^11^ in harsh environment. However, compared to the MWIR window material, the current LWIR window material has a fatal weakness of very poor mechanical performance. Thus, it sets a barrier for this technically important material in practical applications, especially in IR sensing as a transmitting dome in aerobat.

Many researchers have expected to improve and reinforce the mechanical performance of the well-known IR transmitting materials, for example, a thin film with fine mechanical performance is deposited on a IR window material^1^,^10^,^12^. There has been a continuing search for more durable LWIR transmitting materials that can be used as domes in harsh environment^13^,^14^. Efforts, such as material genome project, have been successful to predict useful properties for the known or even unknown inorganic materials^15^,^16^,^17^. Currently, experimental data are often scant for the IR window materials. In fact, to find the potential candidates according to the periodic table is a huge trail-and-error engineering project, which is not practicable in consideration of the limited time and resource. Therefore, this urges a more efficient and economic way to deal with such kind of task of exploring the potential IR transmitting materials. Recently, the rapid development in computer performance and large-scale computing technology has placed the simulation at the forefront to search new materials. Using quantum mechanical techniques, the quantitative predictions on the structure and properties for a material can be archived at a relatively modest economic cost^18^,^19^,^20^. In this work, we present a first-principles gene-like exploration which enables a highly efficient searching for the potential new LWIR transmitting materials with good mechanical performance. Two important descriptors including bulk modulus and the twofold of the max optical vibration frequency are defined and tested to draw a performance map. Seven new potential candidates are selected, namely TiSe, TiS, MgS, CdF_2_, HgF_2_, CdO, and SrO which have not been used before. Especially, the performances of TiS and CdF_2_ are comparable to that of the most popular commercial ZnS at high temperature. The relations between the infrared transmission and the mechanical property are also discussed.

## Results and Discussion

**Figure 1:**
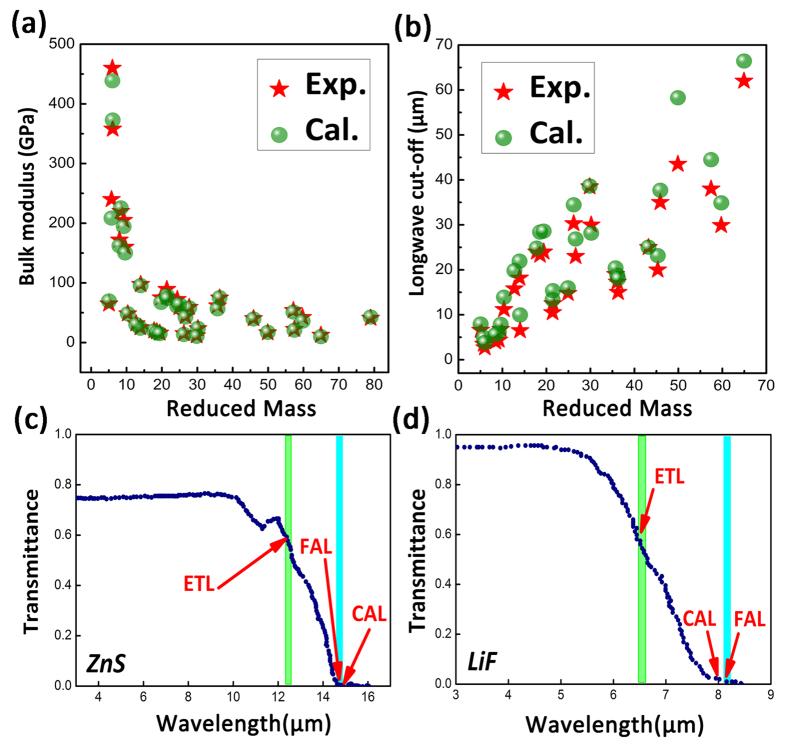
Benchmark test of the theoretical bulk modulus *B*_*v*_ (**a**) and the long-wave absorption limit 
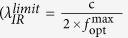
, i.e. calculated absorption limit, CAL) (**b**) for mechanical and optical performance, respectively. Experimental data (bulk modulus and Effective Transmittance Limit, ETL) are collected and compared to the calculated results from ref. 11. The ETL, CAL and FAL (Full Absorption Limit) are shown in the infrared transmittance spectrums of the multispectral ZnS (**c**) and single crystal LiF (**d**) as the example^13^.

First of all, before the large-scale searching, two key descriptors for the optical and the mechanical performance, which are readily calculated, are defined and compared with the experimental data from the current popular transmitting materials. One descriptor is chosen as the Voigt-type bulk modulus *B*_*v*_ to predict mechanical strength^21^,^22^. Another descriptor predicts the IR transmitting ability. In fact, the IR absorption mechanism is intrinsically from the lattice phonon vibration in materials^23^,^24^. Generally, IR window materials with a thickness of several millimeters are normally opaque within the 1-phonon (fundamental) and 2-phonon (overtones) regions^25^. Fairly quantitative predictions can be made in term of the vibration frequencies for the IR transmitting ability: according to White *et al.*^23^,^26^, a good empirical rule-of-thumb to follow is the cutoff of IR transmission occurring at about twice the frequency of the highest optical vibration mode due to the phonon overtone absorption. Absorption coefficients for the 1-phonon fundamental transitions are naturally large in the range 10^3^–10^5^ cm^−1^. Yet, the 2-phonon region in the range of 10^1^–10^3^ cm^−1^ also cannot be neglected. This is the region including the overtones of the fundamental vibrations (e.g. 2TO and 2LO) and the combined bands (e.g. TO+LO, TO+LA)^10^,^27^. However, the coefficient for the 3- or more-phonon region tends to be much smaller, see the details in the supporting information. Therefore, the twofold of the max optical phonon frequency 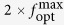 is employed, which generally defines the long-wave absorption limit 
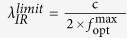
, as another descriptor. Here, *c* is the light speed. In other words, the wavelength at 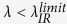 can achieve transmission and be used for window, and that at 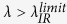 gets the full absorption. Figure 1 summarizes the calculations of the two descriptors for the well-known IR transmitting materials (Thirty-four materials for the mechanical properties and thirty materials for the optical properties) and compares them with experiments^11^. The detailed data can be seen in Table S1 and Table S2 in the supplementary information. It is very clear that the theoretical *B*_*v*_is almost the same as the experimental counterparts except a slight difference. The average discrepancy to the experimental data can be as low as −4.8%. On the other hand, the optical 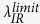 is significantly larger than the experimental data, with the average discrepancy of +20.5%. However, this is not the calculation error but the inconsistent definitions between the reported experimental data and the theoretical cutoff here. To further illustrate the discrepancy, the IR transmittance spectrums of the multispectral zinc sulfide (ZnS) and the crystalline lithium fluoride (LiF) are presented in Fig. 1(c,d) according to ref. 13. The usually reported cutoff of the IR transmitting wavelength in experiment is known as an effective transmittance limit (ETL), which for example employs the absorption coefficient less than 1 cm^−1^ as the criterion^11^. For a sample with a given thickness, an absorption coefficient unit can be converted to a unit of transmittance through Lambert-Beer’s law. The calculated absorption limit (CAL) has the definition according to 
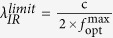
. To provide a useful comparison, another experimental criterion, named as full absorption limit (FAL) (i.e. the minimum wavelength is needed for a full absorption), is also introduced. In fact, the FAL and the CAL agree with each other very well. In other words, the popular used ETL should be intrinsically different from the FAL and thus the CAL. The detailed discussions on the relations among ETL, FAL, and CAL can be found in the supplementary information. We will use the FAL as the standard for the next-step searching. Finally, both the trends with atomic reduced mass are well consistent with experiments^23^. In other words, both the *B*_*v*_ and 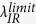 are the reasonable descriptors to search the LWIR window materials.

**Figure 2:**
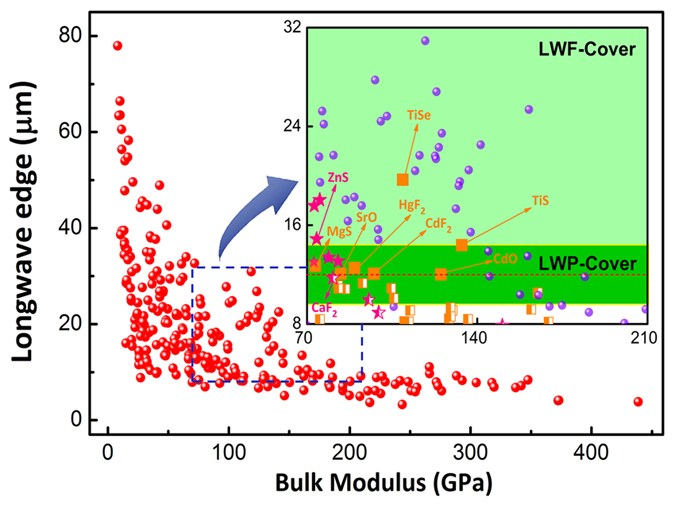
Calculation of the performance map of the 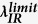 and the *B*_*v*_ for two hundred and fifty-three materials chosen in ICSD. Inset enlarges the key area for the exploration. Here, the regions as noted with LWP-Cover (dark green shading, 9.6~14.5 μm) and with LWF-Cover (bright green shading, >14.5 μm) are for long-wave-partial and long-wave-full transmitting windows by our redefined criteria. The pink stars show the cases of the current popular IR window materials^10^, which demonstrates the division reasonable. The dot line is in the middle of the LWP region. The orange squares highlight the potential infrared window candidates (with notice by orange arrow as well) above the dot line. The samples noted by the purple circles are excluded due to no significant band gap based on the calculations. Other gaped materials below the dot middle line are shown with half filled orange squares or pink stars.

In the next step, the candidates with both the fine optical and mechanical performance for LWIR windows are explored. The procedures are as follow: firstly, the combinations of *A*_*x*_*B*_*y*_ (A for cation and B for anion) are listed according to the periodic table. The simple binary compositions with *x:y* = 1:1, 1:2, 1:3, 2:1, 3:1, 2:3, 3:2, which is easy for future experimental production, are adopted. Secondly, the popular crystal structures for each composition are used. Thirdly, the two descriptors discussed above are calculated. Figure 2 summarizes the performance relation between the *B*_*v*_ and the 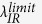 in total two hundred and fifty-three candidates. Table S3 gives all the detailed data. It is very clear that the 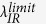 has an inverse trend with the *B*_*v*_. This can be readily understood by the behavior of the simple harmonic vibration,
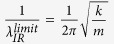
. Here, *k* is the force constant, which reflects the bonding strength in a solid and is positively related to *B*_*v*_. *m* is the reduced mass^2^,^8^. It is well known that a solid with lighter atomic mass holds stronger bond^10^. In other words, lighter-atom solid should have larger bulk modulus as well as higher vibration frequency, thus smaller 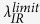, displaying a “banana curve” in Fig. 2. Therefore, there is always a tradeoff between gaining a high LWIR transmission and losing a desirable mechanical property, that’s the reason why the current materials usually cannot meet both the criteria easily.

To evaluate the results, a standard to choose the most possible candidates is defined. Considering the average discrepancy (20.5%) between the ETL and the CAL/FAL by the benchmark test above, the concerned LWIR window (8~12 μm) is accordingly modified to (9.6~14.5 μm), as shown in the inset including LWP (long-wave-partial, 9.6~14.5 μm) and LWF (long-wave-full, >14.5 μm) transmitting window regions. The bulk modulus ≥72 GPa is chosen according to the mechanical performance of the popular ZnS^11^. The pink stars highlight the present used commercial products. For example, ZnS belongs to the LWF-Cover region. That is consistent with its fine transmission of 8~12 μm in experiments^11^. For another example, CaF_2_, with its CAL slightly below the midline (dot line) in the LWP region, has a consistent experimental ETL of 9.4 μm slightly below the midline (10 μm) of 8~12 μm^10^. In other words, most of the commercial materials for IR windows can be also found in our defined “map”.

If only the 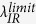 and *B*_*v*_ are used as criteria, it seems substantial materials are available. However, the zero-band-gap material is not suitable for windows due to the feasible IR absorption by their intrinsic free carriers generated at the elevated temperature. Thus, a further filtration is necessary through band structure evaluation for the candidates in LWP and LWF cover regions. Here, to enable a more reasonable prediction of metallic or insulating characteristics, the GGA+U method is applied to the transition-metal compound which has no band gap in a normal GGA calculation^28^,^29^,^30^,^31^,^32^. Here, the materials that show semimetallic or metallic characteristics are excluded (see the purple circles in the inset of Fig. 2). After the selection from the total two hundred and fifty-three *A*_*x*_*B*_*y*_ materials, the orange squares highlight the potential candidates which are suitable for LWIR transmission, namely, TiSe, TiS, MgS, CdF_2_, HgF_2_, CdO and SrO (totally seven candidates). In fact, except TiSe, other candidate shows a larger band gap (>1.7 eV) with further adding 25% *E*_*x*_^*HF*^ in a HSE calculation. According to a rule-of-thumb by Hilton^23^, a material should have a band gap larger than 1.5 eV to suppress the thermal generation of intrinsic free carriers at the elevated temperature. In other words, these candidates (except TiSe) should have the abilities to suppress the absorption of infrared radiation at a relatively high temperature.

**Figure 3:**
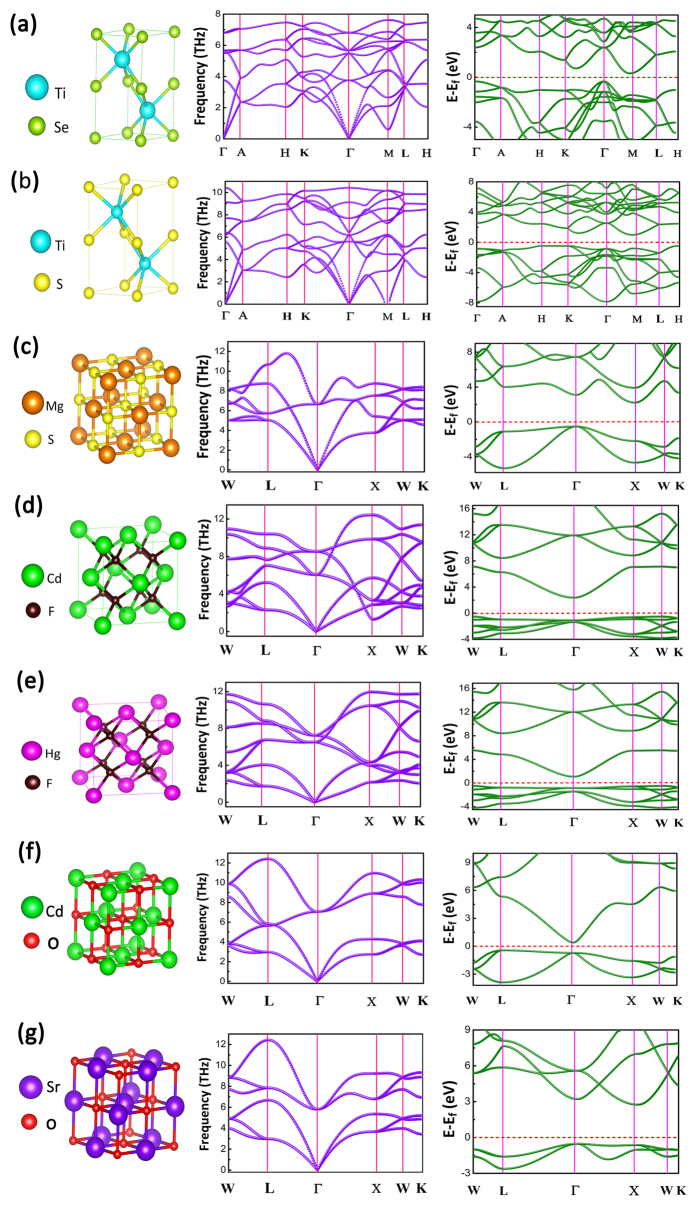
The crystal structures, phonon dispersion and band structures of TiSe (**a**), TiS (**b**), MgS (**c**), CdF_2_ (**d**), HgF_2_ (**e**), CdO (**f**) and SrO (**g**). Color coding: light green for Se, cyan for Ti, yellow for S, orange for Mg, brown for F, dark green for Cd, pink for Hg, red for O and purple for Sr. The Fermi Level is marked with red dashed lines in band structures.

Stability is another important factor for the application of the new potential candidates. The crystal structures, phonon dispersion and band structures of TiSe, TiS, MgS, CdF_2_, HgF_2_, CdO, and SrO are displayed in Fig. 3. The dynamic stabilities for all the seven candidates are clearly demonstrated without any imaginary frequency. All these candidates hold significant electronic band gap. These show their stability at relatively low temperature.

**Figure 4:**
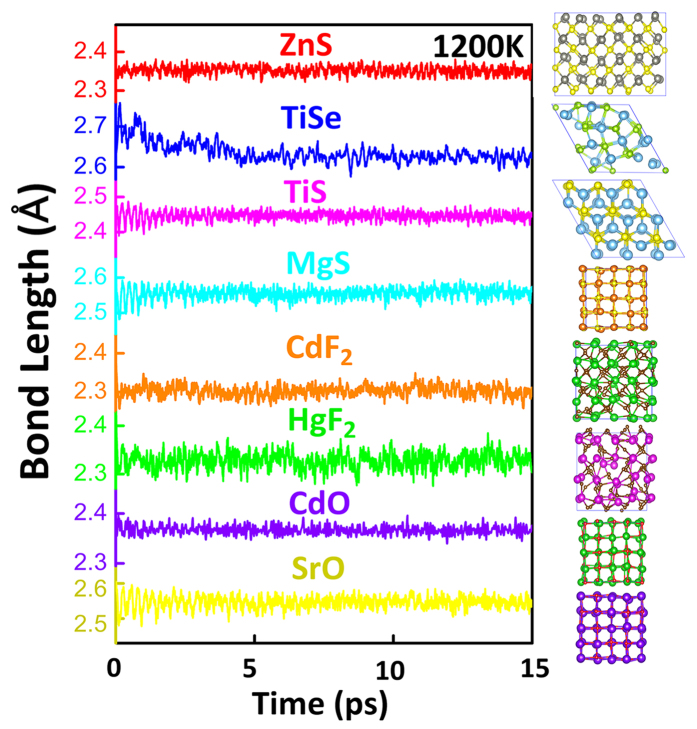
Bond-length evolutions of TiSe, TiS, MgS, CdF_2_, HgF_2_, CdO and SrO in 15-ps molecular dynamics at 1200 K. The final snapshot of the crystal structure after 15-ps annealing is shown together with its bond-length evolution.

To further examine the applicability of these IR candidates in harsh external environment such as at the very high temperature, molecular dynamic simulations are carried out for these compounds. High-temperature molecular dynamics (HTMD) methods are widely used to test the thermal stability and behavior in materials^33^. Especially, here, ZnS is also evaluated to compare with the selected LWIR-window candidates. The temperature is set up to 1200 K to mimic the experimental high temperature^1^,^10^. After a 15-ps *NVT* simulation, the snapshots of each crystal structure for TiSe, TiS, MgS, CdF_2_, HgF_2_, CdO and SrO are shown in Fig. 4. The evolution of average bond length is analyzed during the high-temperature annealing. As a reference, ZnS shows a relatively good thermal stability with harmonic variation of the bond length. In fact, very little distortive structure after the annealing also supports the stability. In contrast, TiSe and HgF_2_ are seriously distorted after the annealing (see their final structures). Especially, for TiSe, the bond length fluctuates significantly. On the other hand, TiS, MgS, CdF_2_, CdO and SrO have the good thermal stability due to the stable bond-length evolution. After further consideration of chemical activity^34^, for example, SrO and MgS readily react with water and CdO reacts with CO_2_ in the air. Thus, TiS and CdF_2_ should be the most possible candidates for LWIR transmitting windows at high temperature. SrO, MgS and CdO may also work if they are insulated from water and air.

**Figure 5:**
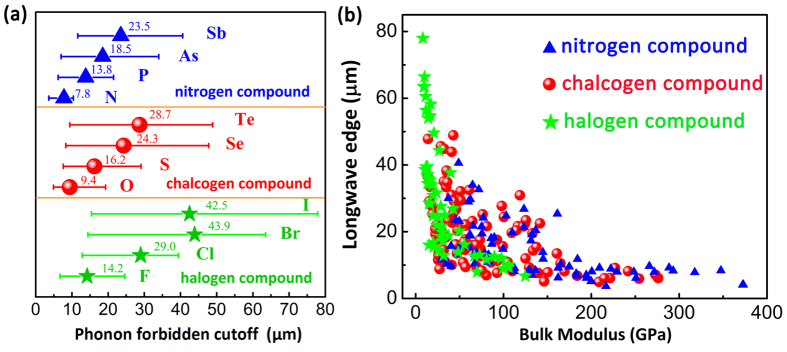
(**a**) Statistic average value of the long-wave absorption limit 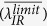 for halogen, chalcogen, and nitrogen compound. Both the minimum and maximum are shown to see the range of the 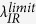. (**b**) The Corresponding “banana” performance curve for the three kinds of compounds.

Last but not least, we try to summarize the “gene” behaviour of the materials towards the IR window application and tell the possible design rule. Figure 5(a) shows the average long-wave absorption limit 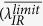, i.e. the CAL) for halogen, chalcogen and nitrogen compounds, respectively. For halogen compounds, the average 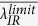 varies from 14.2 to 43.9 μm, which shows the most excellent IR transmission. For chalcogen compounds, the average 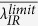 (from 9.4 to 28.7 μm) is shorter but still good for IR windows. However, for nitrogen compounds, the 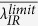 is the smallest from 7.8 to 23.5 μm. In fact, they all obey the “banana” performance curve but just reside in the different regions, see Fig. 5(b). Considering their mechanical performance, chalcogen compounds should be the best platform for IR window candidates. In fact, the IR materials such as ZnS and CdS have been commercially used for IR windows^10^. Most recently, a copolymer with a very good IR transmission has been produced with inverse vulcanization, where molten sulphur, acting as a solvent, was copolymerized with 1,3-diisopropenylbenzene (DIB) to prepare a chemically stable and processable sulphur plastic^35^. Thus, this IR material gene picture may offer some usual references for the future industrial design.

## Conclusions and Outlook

In this work, a simple and efficient method has been proposed to explore the potential LWIR window materials with both the suitable mechanical and optical performances by a first-principles gene-like searching. The two descriptors, namely 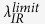 and *B*_*v*_, well describe the properties for IR window materials. Especially, in the benchmark test, the theoretical simulations are well consistent with experiments. From two hundred and fifty-three binary compounds with *A*_*x*_*B*_*y*_ form, seven new potential candidates of TiSe, TiS, MgS, CdF_2_, HgF_2_, CdO, and SrO are finally decided. Especially, the performances of TiS and CdF_2_ can be comparable to the most popular ZnS at high temperature and in chemical stability. The intrinsic “gene” behaviour of the IR material is also studied to facilitate the future search for IR window materials. Here, we stress that the development of a practical IR window is in fact a big engineering project. To meet the requirement, people must carefully control the defects during the growth of material because defects or unintentional impurities can always lead to IR absorptions due to the local state inside the band gap or the free carrier supplement at band edge. Also because some large scale defects (such as boundary, void or dislocation) can scatter IR seriously, thus worsen the transmission. On the other hand, some cover layers are also required to protect the IR window materials from the erosion by wind and rain. At this stage, we still have not considered the problems mentioned above, however the present strategy at least tells people which candidate should be focused on for IR window materials. We believe the same idea can also be used in searching other materials toward a special performance with the help of material gene-like exploration.

## Methods

Phonon dispersion and elastic constants are obtained to estimate the long-wave absorption limit 
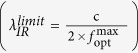
 and bulk modulus for two hundred and fifty-three kinds of *A*_*x*_*B*_*y*_ binary compounds, based on first-principles calculations. The theoretical investigations employ the density functional theory^36^,^37^ and Vienna *Ab-initio* Simulations Package (VASP)^38^,^39^. The projector augmented wave (PAW) pseudopotentials^40^ are used to describe electron-ion interactions. For the exchange-correlation energies between electrons, the Perdew-Burke-Ernzerhof (PBE) functional^41^ is used. The energy cutoff for the plane wave expansion is chosen equal to or larger than 1.3 folds of the suggested maximum. Lattice geometry relaxation is run until all the forces are smaller than 0.01 eV/Å. Most of crystal cells employ Monkhorst-Pack 9 × 9 × 9 mesh in Brillouin zone integration. The candidates are chosen in form of *A*_*x*_*B*_*y*_ (with *x:y* = 1:1, 1:2, 1:3, 2:1, 3:1, 2:3 and 3:2) in inorganic crystal structure database (ICSD). Here, the phonon dispersion calculation uses the supercell method by phonopy code^42^. The supercell is chosen empirically with either 2 × 2 × 2, or 3 × 3 × 3, or 4 × 4 × 4 folds of the primitive cell for different materials to meet the precision of the calculation (with lattice cell size ≥10 Å). Bulk modulus *B*_*v*_ (Voigt-type) for cubic, hexagonal, or tetragonal lattice is calculated from the elastic constant (*C*_*ij*_) by Voigt-Reuss-Hill approximations^43^. High-temperature molecular dynamics are run at 1200 K for 15 ps to test the thermal stability of the selected LWIR-window candidates. The time step is 1 fs and the canonical *NVT* ensemble is used^44^. Only Γ point in the Brillouin zone sampling is chosen for molecular dynamics calculations. To reasonably describe the electronic band structure, the GGA+U method is applied to the transition-metal compound which has no band gap in a normal GGA calculation.

## Additional Information

**How to cite this article**: Du, J.-R. *et al.* Exploring long-wave infrared transmitting materials with *A_x_B_y_* form: First-principles gene-like studies. *Sci. Rep.* **6**, 21912; doi: 10.1038/srep21912 (2016).

## Supplementary Material

Supplementary Information
