# Supplementary material for: Exploring long-wave infrared transmitting materials with AxBy form: First-principles gene-like studies
Source: Sci Rep. 2016 Feb 23;6:21912. doi: 10.1038/srep21912 (PMC4763250; doi:10.1038/srep21912)
Supplement: Supplementary Information [file srep21912-s1.pdf]

Supporting Information for

Exploring long-wave infrared transmitting materials with A_xB_y form: First-principles gene-like studies

Jia-Ren Du, Nian-Ke Chen, Xian-Bin Li,* Sheng-Yi Xie, Wei Quan Tian, Xian-Yin Wang, Hai-Ling Tu, and Hong-Bo Sun*

Table S1. Benchmark test of the theoretical bulk modulus B_v for mechanical performance. Experimental data are collected and compared from Reference[12].

Materials	Bulk Modulus (GPa) Exp.	Bulk Modulus (GPa) Cal.	Materials	Bulk Modulus (GPa) Exp.	Bulk Modulus (GPa) Cal.
AlAs	75.3	67.40	SiC	220	225.67
BP	172	162.14	AgBr	40.6	39.54
GaP	89.3	78.88	CsBr	16.7	17.10
InP	72.5	60.90	KBr	15.2	13.47
BN	358	372.98	NaBr	19.9	19.49
AlN	205	194.46	TlBr	22.4	20.68
BeO	240	208.56	TlCl	23.6	23.67
MgO	160	150.31	NaCl	25.3	24.08
PbS	59.3	58.15	KCl	18.4	16.22
ZnS	76.6	73.93	AgCl	44	42.78
CdS	59	64.13	NaF	48.5	48.27
PbSe	54	51.04	LiF	65	69.77
ZnSe	61.8	56.55	KF	31.8	30.29
CdTe	42.4	35.97	KI	11.9	10.58
PbTe	41.1	42.79	CsI	12.6	10.05
C	460	438.74	NaI	16.1	15.15
Si	97.7	95.44	Ge	75	75.25

Table S2. Benchmark test of the theoretical long-wave absorption limit for optical performance. Experimental data (ETL) are collected and compared from Reference[12].

Materials	Longwave cut-off (µm) Exp.	Longwave cut-off (µm) Cal.	Materials	Longwave cut-off (µm) Exp.	Longwave cut-off (µm) Cal.
GaAs	17.3	18.81	KBr	30.3	34.45
InAs	20	23.14	NaBr	24	24.82
GaP	10.5	13.38	TlBr	38	49.59
AlN	4.4	6.13	TlCl	30	31.65
BeO	3.5	4.97	NaCl	18.2	21.90
MgO	6.8	7.85	KCl	23.3	28.37
ZnS	12.5	14.88	AgCl	23	26.85
CdS	14.8	15.97	NaF	11.2	13.89
ZnSe	19	22.18	LiF	6.6	7.91
CdTe	29.9	34.87	KF	15.8	19.82
ZnTe	25	25.66	KI	38.5	38.63
C	2.7	3.81	CsI	62	66.43
SiC	4	5.56	NaI	24	28.59
AgBr	35	37.68	Si	6.5	9.94
CsBr	43.5	58.25	Ge	15	18.05

Table S3. Calculation of the mechanical and the optical performance in total 253 candidates by first-principles method.

Materials	Space Group	Bulk Modulus (GPa) Cal.	Longwave cut-off (µm) Cal.
BeO	p63mc	208.56	4.97
MgO	p63mc	139.47	7.65
MgO	fm3m	150.31	7.85
CaO	p63mc	85.78	10.83
CaO	fm3m	104.65	10.88
SrO	fm3m	83.93	12.07
BaO	fm3m	69.38	13.65
ZnO	f43m	129.88	9.09
ZnO	p63mc	129.06	9.32
ZnO	fm3m	164.87	10.52
CdO	fm3m	125.03	12.01
CdO	p63mc	92.88	11.29
NbO	pm3m	241.79	8.19
NaS	p63mmc	25.56	11.56
BeS	f43m	92.62	7.69
MgS	fm3m	73.41	12.69
MgS	f43m	55.92	14.58
CaS	fm3m	56.53	17.67
SrS	fm3m	48.15	20.82

**BaS**	**fm3m**	**41.40**	**22.69**
**GaS**	**p63mmc**	**26.19**	**13.84**
**PbS**	**fm3m**	**58.15**	**21.87**
**ScS**	**fm3m**	**99.20**	**15.65**
**YS**	**fm3m**	**92.49**	**17.58**
**TiS**	**p63mmc**	**133.65**	**14.38**
**CoS**	**p63mmc**	**160.72**	**13.49**
**NiS**	**p63mmc**	**137.32**	**15.43**
**ZnS**	**f43m**	**73.93**	**14.88**
**ZnS**	**p63mc**	**69.28**	**15.02**
**CdS**	**fm3m**	**72.97**	**17.57**
**CdS**	**p63mc**	**64.13**	**15.97**
**CdS**	**f43m**	**54.91**	**16.95**
**HgS**	**f43m**	**68.46**	**16.09**
**NaSe**	**p63mmc**	**22.29**	**20.93**
**BeSe**	**f43m**	**75.32**	**8.37**
**MgSe**	**f43m**	**45.56**	**16.23**
**MgSe**	**fm3m**	**60.14**	**15.62**
**CaSe**	**fm3m**	**47.55**	**20.32**
**SrSe**	**fm3m**	**40.70**	**31.02**
**BaSe**	**fm3m**	**35.55**	**37.95**
**PbSe**	**fm3m**	**51.04**	**32.11**
**ScSe**	**fm3m**	**80.92**	**21.66**
**YSe**	**fm3m**	**76.24**	**25.23**
**TiSe**	**p63mmc**	**109.37**	**19.67**
**CoSe**	**p63mmc**	**132.89**	**19.53**
**NiSe**	**p63mmc**	**116.28**	**21.65**
**RhSe**	**p63mmc**	**141.43**	**22.51**
**AgSe**	**f43m**	**51.31**	**29.42**
**ZnSe**	**p63mc**	**56.55**	**21.82**
**ZnSe**	**f43m**	**56.55**	**22.18**
**CdSe**	**p63mc**	**45.76**	**26.65**
**CdSe**	**f43m**	**45.99**	**26.88**
**HgSe**	**f43m**	**43.58**	**29.18**
**BeTe**	**f43m**	**56.62**	**9.46**
**MgTe**	**f43m**	**34.14**	**18.27**
**MgTe**	**p63mc**	**34.02**	**18.59**
**CaTe**	**fm3m**	**37.11**	**23.37**
**SrTe**	**fm3m**	**32.03**	**35.04**
**BaTe**	**fm3m**	**28.66**	**45.61**
**BaTe**	**pm3m**	**32.50**	**44.82**
**InTe**	**fm3m**	**40.96**	**43.94**
**PbTe**	**fm3m**	**42.79**	**48.91**
**AsTe**	**fm3m**	**61.02**	**32.52**

**ScTe**	**p63mmc**	**64.52**	**23.18**
**MnTe**	**p63mmc**	**124.21**	**22.30**
**FeTe**	**p63mmc**	**122.65**	**21.60**
**CoTe**	**p63mmc**	**114.47**	**20.40**
**RhTe**	**p63mmc**	**125.44**	**23.44**
**NiTe**	**p63mmc**	**100.37**	**24.41**
**PdTe**	**p63mmc**	**98.01**	**27.75**
**PtTe**	**p63mmc**	**118.63**	**30.93**
**ZnTe**	**p63mc**	**43.01**	**25.78**
**ZnTe**	**f43m**	**42.94**	**25.66**
**CdTe**	**p63mc**	**34.68**	**33.38**
**CdTe**	**f43m**	**35.97**	**34.87**
**HgTe**	**f43m**	**35.06**	**38.32**
**BN**	**p63mc**	**372.30**	**4.08**
**BN**	**p63mmc**	**216.66**	**3.69**
**BN**	**f43m**	**372.98**	**4.13**
**AlN**	**p63mc**	**194.46**	**6.13**
**AlN**	**f43m**	**194.18**	**7.04**
**AlN**	**fm3m**	**250.80**	**6.13**
**GaN**	**p63mc**	**183.59**	**6.83**
**GaN**	**f43m**	**182.81**	**7.42**
**GaN**	**fm3m**	**229.41**	**7.98**
**InN**	**f43m**	**128.36**	**8.44**
**InN**	**p63mc**	**129.11**	**8.60**
**InN**	**fm3m**	**174.88**	**9.51**
**ScN**	**fm3m**	**200.49**	**8.07**
**YN**	**fm3m**	**162.21**	**9.17**
**YN**	**f43m**	**112.38**	**9.09**
**YN**	**p63mc**	**136.20**	**8.39**
**TiN**	**fm3m**	**293.15**	**8.31**
**ZrN**	**fm3m**	**266.04**	**9.69**
**ZrN**	**p63mc**	**222.19**	**8.90**
**HfN**	**fm3m**	**288.46**	**9.22**
**NbN**	**p63mmc**	**317.71**	**7.83**
**TaN**	**p63mmc**	**347.46**	**8.38**
**FeN**	**f43m**	**271.70**	**7.94**
**CoN**	**f43m**	**248.42**	**7.97**
**NiN**	**f43m**	**209.24**	**9.19**
**CuN**	**f43m**	**165.11**	**10.33**
**BP**	**f43m**	**162.14**	**6.23**
**AlP**	**p63mc**	**82.81**	**11.11**
**AlP**	**f43m**	**82.98**	**10.87**
**GaP**	**fm3m**	**89.48**	**18.27**
**GaP**	**p63mc**	**78.39**	**13.36**

**GaP**	**f43m**	**78.88**	**13.38**
**InP**	**f43m**	**60.90**	**15.30**
**InP**	**fm3m**	**75.42**	**19.46**
**SiP**	**f43m**	**69.38**	**13.46**
**SnP**	**fm3m**	**75.01**	**21.54**
**ScP**	**fm3m**	**99.36**	**14.82**
**YP**	**fm3m**	**86.82**	**16.34**
**TiP**	**p63mmc**	**145.04**	**11.85**
**ZrP**	**fm3m**	**144.57**	**13.88**
**VP**	**p63mmc**	**184.27**	**11.81**
**BAs**	**f43m**	**132.32**	**7.01**
**AlAs**	**p63mc**	**67.07**	**13.60**
**AlAs**	**p63mmc**	**82.76**	**13.08**
**AlAs**	**f43m**	**67.40**	**13.28**
**GaAs**	**p63mc**	**61.71**	**19.17**
**GaAs**	**f43m**	**62.29**	**18.81**
**InAs**	**fm3m**	**62.15**	**29.71**
**InAs**	**f43m**	**49.60**	**23.14**
**SnAs**	**fm3m**	**64.73**	**34.00**
**ScAs**	**fm3m**	**85.92**	**18.05**
**YAs**	**fm3m**	**76.91**	**24.18**
**ZrAs**	**fm3m**	**123.01**	**21.38**
**NiAs**	**p63mmc**	**131.23**	**17.3**
**AlSb**	**f43m**	**49.64**	**15.24**
**GaSb**	**f43m**	**45.45**	**23.57**
**InSb**	**f43m**	**37.21**	**30.04**
**InSb**	**fm3m**	**48.80**	**40.61**
**ScSb**	**fm3m**	**65.28**	**21.74**
**YSb**	**fm3m**	**60.04**	**28.98**
**FeSb**	**p63mmc**	**136.49**	**20.46**
**CoSb**	**p63mmc**	**132.37**	**19.19**
**IrSb**	**p63mmc**	**161.24**	**25.37**
**NiSb**	**p63mmc**	**109.82**	**19.80**
**PdSb**	**p63mmc**	**102.87**	**24.82**
**PtSb**	**p63mmc**	**123.23**	**26.80**
**C**	**p63mmc**	**243.51**	**3.26**
**C**	**fd3ms**	**438.74**	**3.81**
**Si**	**fd3ms**	**95.44**	**9.94**
**Ge**	**fd3ms**	**75.25**	**18.05**
**Sn**	**fd3ms**	**36.60**	**28.37**
**SiC**	**f43m**	**225.67**	**5.56**
**ScC**	**fm3m**	**157.61**	**10.37**
**TiC**	**fm3m**	**265.99**	**11.08**
**ZrC**	**fm3m**	**237.76**	**7.47**

**HfC**	**fm3m**	**255.67**	**6.83**
**VC**	**fm3m**	**319.48**	**6.90**
**NbC**	**fm3m**	**304.74**	**7.83**
**TaC**	**fm3m**	**341.90**	**7.19**
**CrC**	**fm3m**	**337.39**	**6.79**
**ZrB**	**fm3m**	**169.73**	**9.42**
**HfB**	**fm3m**	**185.90**	**8.94**
**LiF**	**fm3m**	**69.77**	**7.91**
**NaF**	**fm3m**	**48.27**	**13.89**
**KF**	**pm3m**	**37.40**	**18.79**
**KF**	**fm3m**	**30.29**	**19.82**
**RbF**	**pm3m**	**31.00**	**17.33**
**RbF**	**fm3m**	**29.67**	**21.71**
**CsF**	**pm3m**	**28.83**	**18.57**
**CsF**	**fm3m**	**24.74**	**24.46**
**LiCl**	**fm3m**	**31.73**	**12.86**
**NaCl**	**fm3m**	**24.08**	**21.90**
**KCl**	**pm3m**	**19.76**	**30.90**
**KCl**	**fm3m**	**16.22**	**28.37**
**RbCl**	**pm3m**	**16.32**	**34.55**
**RbCl**	**fm3m**	**13.80**	**37.09**
**CsCl**	**pm3m**	**14.33**	**36.00**
**CsCl**	**fm3m**	**11.76**	**39.45**
**TlCl**	**pm3m**	**23.67**	**31.65**
**TlCl**	**fm3m**	**18.79**	**34.01**
**AgCl**	**fm3m**	**42.78**	**26.85**
**CuCl**	**f43m**	**49.67**	**20.22**
**LiBr**	**fm3m**	**25.64**	**14.44**
**NaBr**	**fm3m**	**19.49**	**24.82**
**KBr**	**pm3m**	**16.34**	**35.46**
**KBr**	**fm3m**	**13.47**	**34.45**
**RbBr**	**fm3m**	**11.62**	**56.37**
**RbBr**	**pm3m**	**14.19**	**54.02**
**CsBr**	**fm3m**	**10.20**	**63.51**
**CsBr**	**pm3m**	**17.10**	**58.25**
**AgBr**	**fm3m**	**39.54**	**37.68**
**TlBr**	**pm3m**	**20.68**	**49.59**
**TlBr**	**fm3m**	**16.48**	**54.76**
**LiI**	**fm3m**	**19.80**	**16.74**
**LiI**	**p63mmc**	**20.28**	**15.44**
**LiI**	**p63mc**	**15.05**	**16.01**
**NaI**	**fm3m**	**15.15**	**28.59**
**KI**	**pm3m**	**13.28**	**39.47**
**KI**	**fm3m**	**10.58**	**38.63**

**RbI**	**pm3m**	**11.41**	**60.56**
**RbI**	**fm3m**	**9.13**	**63.43**
**CsI**	**fm3m**	**8.17**	**77.97**
**CsI**	**pm3m**	**10.05**	**66.43**
**AgI**	**f43m**	**26.78**	**44.29**
**Mg_2Ge**	**fm3m**	**49.66**	**15.49**
**Mg_2Si**	**fm3m**	**54.51**	**15.14**
**Ag_2O**	**pn3ms**	**68.73**	**9.74**
**Cu_2O**	**pn3ms**	**110.02**	**8.21**
**K_2O**	**fm3m**	**27.05**	**17.54**
**Li_2O**	**fm3m**	**80.52**	**6.96**
**Na_2O**	**fm3m**	**46.92**	**13.52**
**Rb_2O**	**fm3m**	**22.55**	**19.31**
**K_2Se**	**fm3m**	**16.65**	**33.42**
**Li_2Se**	**fm3m**	**33.63**	**11.03**
**Na_2Se**	**fm3m**	**24.50**	**22.24**
**Rb_2Se**	**fm3m**	**13.99**	**47.80**
**K_2S**	**fm3m**	**18.81**	**27.56**
**Li_2S**	**fm3m**	**40.91**	**10.04**
**Na_2S**	**fm3m**	**28.94**	**20.47**
**Rb_2S**	**fm3m**	**15.60**	**29.18**
**K_2Te**	**fm3m**	**13.71**	**36.61**
**Li_2Te**	**fm3m**	**26.05**	**12.89**
**Na_2Te**	**fm3m**	**19.30**	**25.34**
**Li_3As**	**p63mmc**	**35.16**	**10.38**
**Li_3P**	**p63mmc**	**40.24**	**9.73**
**Li_3Sb**	**p63mmc**	**28.84**	**11.64**
**Li_3Sb**	**fm3m**	**32.55**	**12.15**
**AlF_3**	**r3h**	**124.59**	**6.71**
**NbF_3**	**pm3m**	**105.64**	**9.40**
**$BaCl_2$**	**fm3m**	**28.63**	**27.63**
**$SrCl_2$**	**fm3m**	**33.20**	**24.99**
**MoS_2**	**p63mmc**	**53.96**	**10.84**
**BaF_2**	**fm3m**	**55.73**	**16.10**
**CaF_2**	**fm3m**	**80.63**	**11.72**
**CdF_2**	**fm3m**	**97.50**	**12.06**
**HgF_2**	**fm3m**	**89.54**	**12.54**
**MgF_2**	**p42mnm**	**99.52**	**8.92**
**PbF_2**	**fm3m**	**72.82**	**13.04**
**SrF_2**	**fm3m**	**66.85**	**12.76**
**ZnF_2**	**p42mnm**	**105.17**	**10.07**
**GeO_2**	**p42mnm**	**221.30**	**6.12**
**SiO_2**	**p42mnm**	**146.26**	**5.12**
**SnO_2**	**p42mnm**	**183.94**	**6.83**

**TiO_2**	**p42mnm**	**224.13**	**6.08**
**SiS_2**	**I42d**	**26.00**	**8.83**
**ZrS_2**	**p3m1**	**32.27**	**14.57**
**$AuSb_2$**	**pa-3**	**71.80**	**32.67**
**Al_2O_3**	**R3CR**	**254.29**	**5.94**
**Al_2O_3**	**R3CH**	**276.11**	**6.11**
**Ga_2O_3**	**R3CR**	**273.04**	**7.11**
**In_2O_3**	**R3CH**	**169.37**	**8.16**
**Ti_2O_3**	**R3CR**	**227.04**	**9.15**
**Be_3N_2**	**Ia3**	**199.79**	**5.13**
**Ca_3N_2**	**Ia3**	**67.04**	**10.41**
**Mg_3As_2**	**Ia3**	**40.21**	**15.68**
**Mg_3N_2**	**Ia3**	**111.87**	**8.18**
**Mg_3P_2**	**Ia3**	**47.76**	**13.39**

Discussion on the Relations among ETL, FAL, and CAL

Usually, in window material, long-wave IR absorption intrinsically comes from its lattice vibration. Experimental studies on a wide variety of solids with band gap have demonstrated that, the absorption coefficient α decreases nearly exponentially with the frequency ω in the range of $\omega_g \gg \omega \gg \omega_o$

$$\alpha \approx A \exp(-B\, \omega/\omega_o)$$

Here, ω_g is the frequency corresponding to its band gap, ω_o is the frequency corresponding to its optical phonon. A is about 10^4-10^5 and B is about 4-5 in most solids [Annual Review of Materials Science 7, 23 (1977)].

Such exponential decrease of α vs. ω can be understood in terms of multiphonon absorption process, i.e. for $\omega > \omega_o$, just $\omega = m \times \omega_o$ (m= 2, 3, 4 …) can be absorbed. In the paper, we define the "full absorption limit (FAL)" of frequency, in which the absorption coefficient is approximately 10^2 cm^{-1}, see Fig. S1. In fact, in general, considering a typical thickness of the IR window (L ~ 0.5 cm), such α is corresponding to almost no any transmittance ($T = e^{-50}$) according to Lambert–Beer law (T=$e^{-\alpha \times L}$). However, when α drops from 10^2 cm^{-1} to 1 cm^{-1}, the transmittance changes significantly from 0 % to 60 %. As such, people like to choose a typical absorption coefficient, for example $\alpha =$ 1 cm^{-1}, as the criterion for effective transmittance limit (ETL), which is used in this paper. In other words, FAL and ETL are different experimental standards to define the start of IR transmission. FAL starts from 0 % transmittance while ETL starts from an effective transmittance (such as 60 %). Coincidently, the two-phonon frequency ($\omega = 2 \times \omega_o$) is usually well

consistent with the FAL, see the green dash line in Fig. S1. Here, the two-phonon frequency is evaluated in the main text as the calculated absorption limits (CAL). As such, the CAL is corresponding to the experimental FAL but not the ETL.

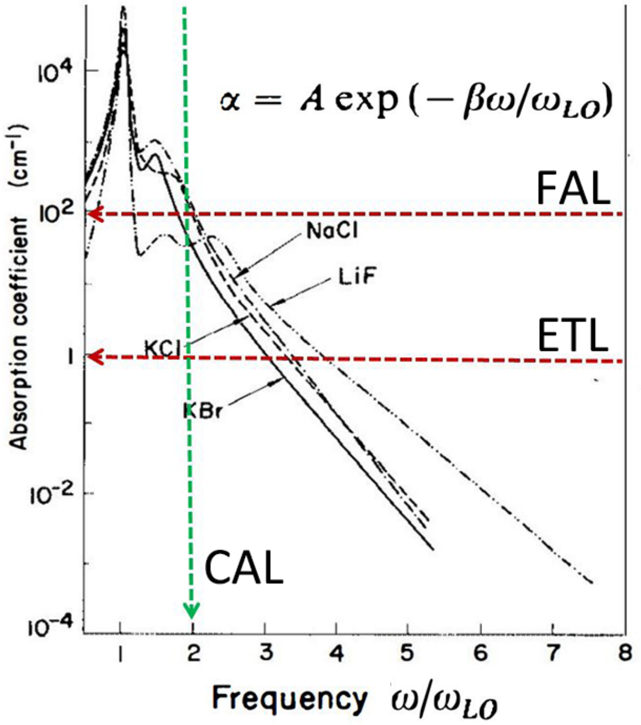

Fig S1. Absorption coefficient vs. frequency for some materials [Ann. Rev. Mater. Sci. 7, 23 (1977)]. FAL and ETL are related to the absorption coefficient of 10^2 and 1 cm^{-1}, respectively. CAL is corresponding to the two-phonon frequency. Copyright 1977 by Annual Reviews Inc.
